# Septic Arthritis of the Manubriosternal Joint in an Immunocompetent Adult: A Case Report

**DOI:** 10.7759/cureus.110795

**Published:** 2026-06-13

**Authors:** Yomon Jasim, Mohammad Muneeb, Robert Willington

**Affiliations:** 1 Medicine, University Hospitals Dorset National Health Service (NHS) Foundation Trust, Bournemouth, GBR; 2 Acute Medicine, University Hospitals Dorset National Health Service (NHS) Foundation Trust, Bournemouth, GBR

**Keywords:** case report, chest wall swelling, immunocompetent, septic arthritis, manubriosternal joint

## Abstract

Septic arthritis of the manubriosternal joint (MSJ) is an exceptionally rare clinical entity, particularly in immunocompetent individuals without established predisposing risk factors. Due to its rarity and non-specific presentation, diagnosis is frequently delayed or initially mistaken for more common causes of anterior chest pain and chest wall swelling, including cellulitis, musculoskeletal pain, or cardiopulmonary pathologies.

We report the case of a 71-year-old immunocompetent man who presented with a two-week history of sharp central chest pain and progressive anterior chest wall swelling following a preceding flu-like illness. He had initially been managed in primary care with two courses of oral antibiotics for presumed cellulitis without clinical improvement. On hospital admission, examination demonstrated a tender erythematous swelling over the manubriosternal region with markedly elevated inflammatory markers (C-reactive protein 224 mg/L). Computed tomography (CT) imaging demonstrated inflammatory soft tissue changes both superficial and deep to the manubriosternal joint, raising suspicion for septic arthritis. Although blood cultures were negative. Further investigations, including magnetic resonance imaging (MRI) of the thoracic spine and transthoracic echocardiography, excluded alternative infective foci. Following multidisciplinary discussion involving acute medicine, microbiology, radiology, and cardiothoracic surgery teams, the patient was managed conservatively with prolonged intravenous and oral antibiotic therapy, resulting in significant clinical, biochemical, and radiological improvement over four weeks without surgical intervention.

This case highlights the diagnostic challenges associated with this rare condition and underscores the importance of early imaging, multidisciplinary assessment, and prompt antimicrobial therapy in achieving favourable outcomes.

## Introduction

The manubriosternal joint (MSJ) is a secondary cartilaginous symphysis between the inferior border of the manubrium and the superior aspect of the sternal body. It allows only minimal movement, primarily facilitating subtle thoracic expansion during respiration. Owing to its fibrocartilaginous structure and lack of a synovial lining, septic involvement of this articulation is very uncommon. 

Septic arthritis most frequently affects large synovial joints such as the knee, hip, and shoulder, while axial or anterior chest wall involvement remains rare. The reported risk factors for septic arthritis include immunosuppression, diabetes mellitus, intravenous drug use, rheumatoid arthritis, recent trauma, and bacteraemia [1-5]. Septic arthritis of the MSJ has rarely been described in the literature, particularly in immunocompetent individuals [1-4].

The diagnosis may be challenging because the presenting symptoms frequently mimic more common causes of chest pain, including musculoskeletal pathology, cellulitis, pulmonary embolism, acute coronary syndrome, costochondritis, and soft tissue infection. Early recognition is important to prevent complications, including joint destruction, osteomyelitis, abscess formation, and mediastinal extension [1-4].

We present a case of presumed septic arthritis of the manubriosternal joint in an immunocompetent adult successfully managed with conservative antimicrobial therapy alone.

## Case presentation

A 71-year-old man presented with a two-week history of sharp central chest pain associated with progressive swelling over the anterior chest wall. The symptoms developed following a preceding flu-like illness. The pain was constant, sharp, severe in intensity, radiated towards the axilla, and was exacerbated by coughing and deep inspiration.

He was initially reviewed in primary care, where a presumptive diagnosis of cellulitis was made. He subsequently received two courses of oral antibiotics, consisting of flucloxacillin 500 mg four times daily for five days, followed by clarithromycin 500 mg twice daily for a further five days. Despite treatment, the symptoms failed to improve. While awaiting outpatient ultrasonography of the chest wall swelling, he developed pyrexia and worsening constitutional symptoms, prompting emergency hospital admission.

His past medical history included haemochromatosis (not requiring active treatment), gout, glaucoma, hypertension, and ischaemic heart disease. There was no history of diabetes mellitus, rheumatoid arthritis, immunosuppressive therapy, or intravenous drug use.

On examination, he was noted to have a tender erythematous swelling overlying the manubriosternal region. Given focal tenderness over the thoracic spine, concern was raised regarding possible vertebral infection.

Initial laboratory investigations demonstrated a marked inflammatory response (Table 1).

**Table 1:** Initial laboratory investigations CRP: C-reactive protein; WBC: white blood cell; eGFR: estimated glomerular filtration rate.

Parameter	Result	Normal Range
CRP	224 mg/L	0-4.9 mg/L
WBC	7.3x10^9^/L	4-10x10*9/L
Neutrophil count	4.9x10^9^/L	2-7x10*9/L
Serum urea level	2.5 mmol/L	2.5-6.7 mmol/L
Serum creatinine	71 µmol/L	95-104 µmol/L
eGFR	>90 mL/min/1.73m²	>90 mL/min/1.73m²

Chest radiography demonstrated clear lung fields without pleural or other acute cardiopulmonary abnormality (Figure 1).

**Figure 1:**
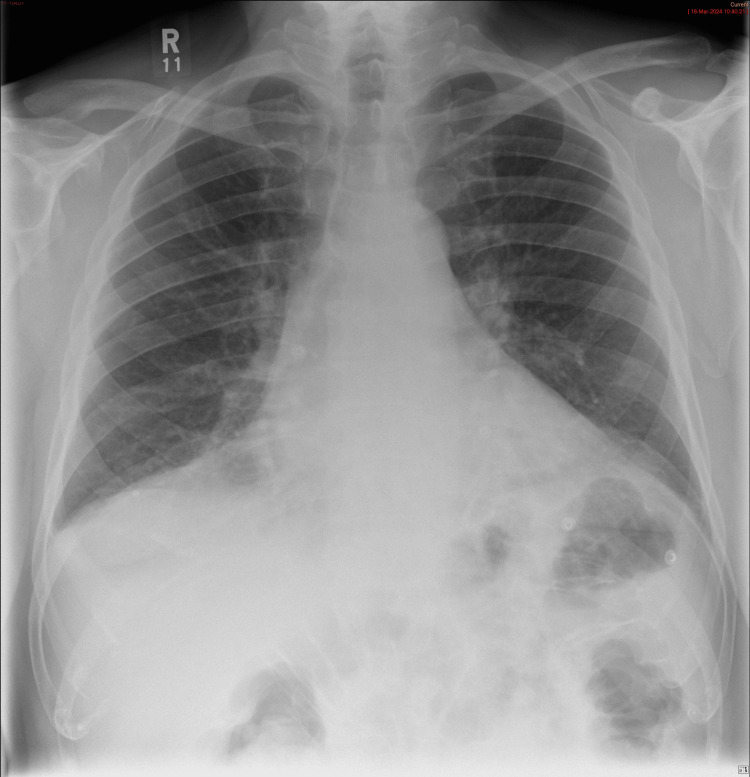
Initial chest radiograph demonstrating clear lung fields with no acute cardiopulmonary abnormality. No MSK abnormality noted. MSK: Musculoskeletal

Contrast-enhanced CT of the chest demonstrated soft tissue inflammatory changes both superficial and deep to the manubriosternal joint, raising suspicion for an infective process involving the joint (Figure 2).

**Figure 2:**
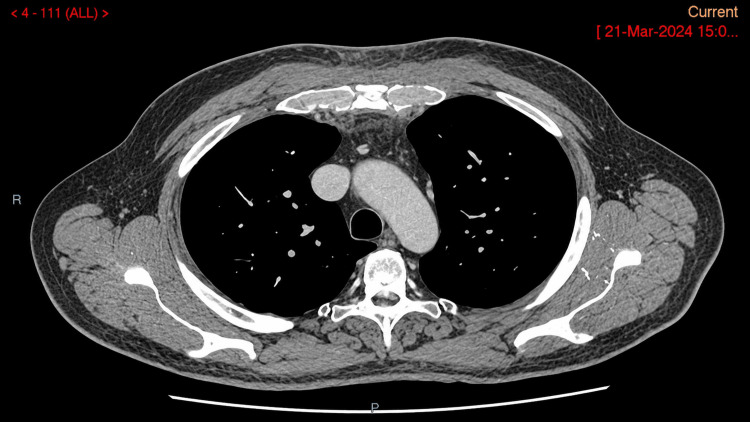
Initial contrast-enhanced CT chest demonstrating inflammatory soft tissue swelling both superficial and deep to the manubriosternal joint, suggestive of inflammatory process.

Blood cultures obtained during admission were negative. Given the unusual anatomical location and clinical concern for a deeper infective focus, multidisciplinary discussion was undertaken involving microbiology and cardiothoracic surgery. Empirical antimicrobial therapy was escalated to intravenous ceftriaxone 2 g once daily.

Because the patient also reported focal tenderness over the T8 vertebral level, MRI of the thoracic spine was performed to exclude vertebral osteomyelitis or discitis. MRI suggested no evidence of discitis, osteomyelitis, paravertebral infection, or other spinal pathology (Figure 3).

**Figure 3:**
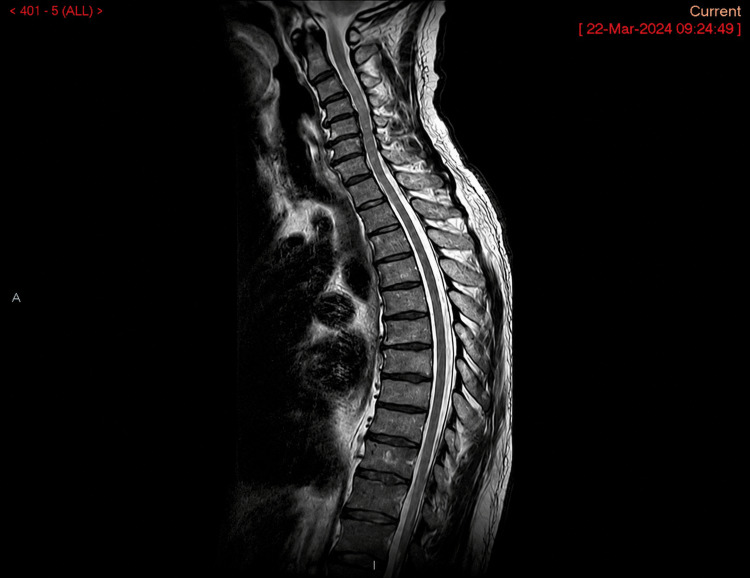
MRI thoracic spine demonstrating no evidence of discitis, vertebral osteomyelitis, or paravertebral soft tissue infection.

Transthoracic echocardiography was performed to assess for infective endocarditis as a potential source of haematogenous spread; but no valvular vegetations or other features of infective endocarditis were identified. Following cardiothoracic surgical review, conservative management with antimicrobial therapy and interval imaging surveillance was advised and continued.

The patient completed a two-week course of intravenous ceftriaxone administered via a peripherally inserted central catheter (PICC), followed by oral co-amoxiclav 625 mg three times daily for a further two weeks. Repeat CT imaging performed after one week demonstrated significant improvement in the previously identified inflammatory soft tissue changes around the manubriosternal joint (Figure 4), suggesting a good response to treatment.

**Figure 4:**
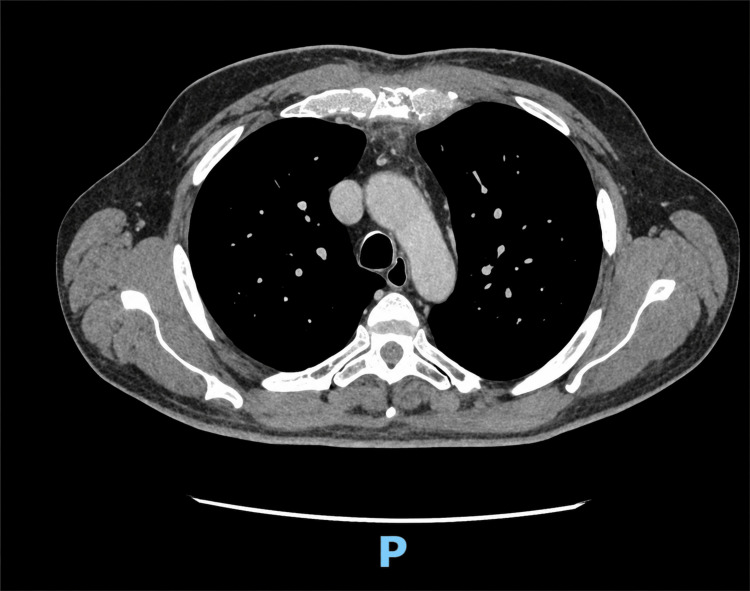
Interval CT chest after one week of intravenous antimicrobial therapy demonstrating significant improvement in inflammatory changes surrounding the manubriosternal joint.

At six-week follow-up, the patient reported a marked symptomatic improvement. Repeat CT imaging demonstrated substantial reduction in soft-tissue inflammatory change, although destructive bony changes across the manubriosternal articulation remained evident, consistent with interval joint destruction secondary to the infective process (Figure 5). C-reactive protein (CRP) had improved to 20 mg/L.

**Figure 5:**
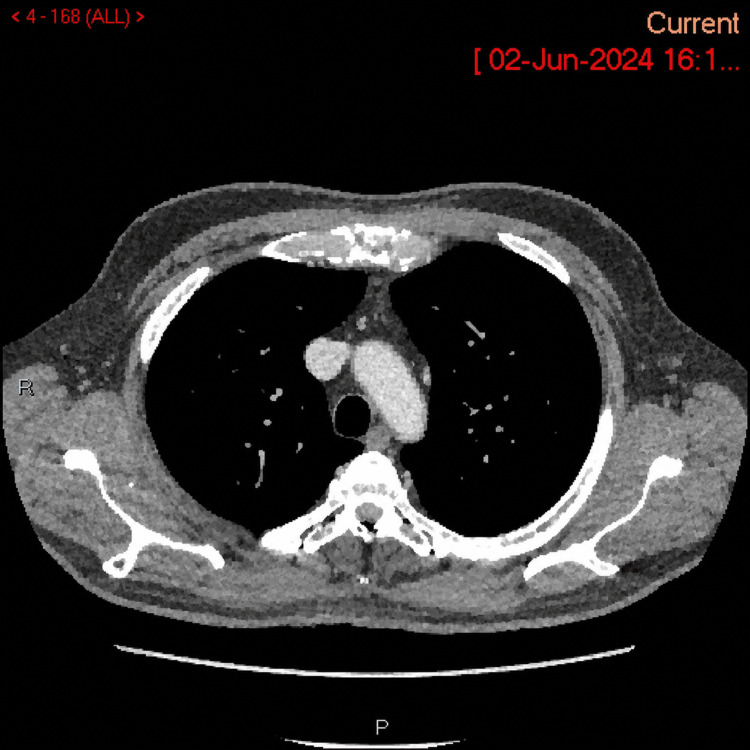
Follow-up CT chest at six weeks demonstrating marked reduction in surrounding soft tissue inflammation with persistent destructive osseous changes involving the manubriosternal joint.

Overall, the patient demonstrated significant clinical, biochemical, and radiological recovery without requiring surgical intervention.

## Discussion

Septic arthritis of the manubriosternal joint is exceptionally rare, particularly in immunocompetent adults [1-4]. The most reported cases involve identifiable predisposing factors such as immunosuppression, rheumatoid arthritis, diabetes mellitus, trauma, intravenous drug use, or systemic bacteraemia [1-5].

Our patient lacked most conventional risk factors for septic arthritis. Although haemochromatosis may theoretically predispose to infection through altered host immune function and iron metabolism, he was not undergoing active treatment with iron levels within the normal range and was otherwise immunocompetent. This makes the presentation particularly unusual.

Diagnosis of MSJ infection is challenging due to its rarity and the non-specific nature of presenting symptoms [1-4]. Patients often initially present with anterior chest pain, swelling, or tenderness - symptoms more commonly attributed to cellulitis, costochondritis, musculoskeletal chest pain, pulmonary or cardiac causes [1,3,4]. In this case, the initial diagnosis in primary care was presumed cellulitis, resulting in delayed definitive imaging.

Unlike septic arthritis involving large synovial joints, microbiological confirmation may be difficult in atypical chest wall infections, particularly where aspiration is technically challenging or surgical intervention is avoided. In our case, blood cultures were negative, but the diagnosis was supported by a combination of clinical presentation, markedly elevated inflammatory markers, radiological findings suggestive of joint infection, exclusion of alternative infective sources, and clear response to prolonged antimicrobial therapy.

CT imaging proved particularly valuable in identifying inflammatory involvement of the manubriosternal region and monitoring treatment response [1-4]. MRI was useful in excluding spinal infection given focal vertebral tenderness. Echocardiography was also appropriately used to exclude infective endocarditis as a potential source of haematogenous dissemination.

Management of MSJ septic arthritis is not standardised due to its rarity [1-4]. Published reports describe both conservative treatment with prolonged antimicrobial therapy and surgical approaches, including debridement or resection, where abscess formation, extensive osteomyelitis, mediastinal extension, or failure of medical therapy occurs [1-4].

Our patient responded well to medical management alone, avoiding surgical intervention with associated morbidity. However, the interval development of destructive joint changes on follow-up imaging highlights the potentially aggressive local nature of this infection, even in the setting of clinical improvement [2,4].

This case reinforces the importance of maintaining diagnostic suspicion for deep-seated infection in patients presenting with persistent anterior chest wall pain and swelling, especially when initial treatment for superficial infection fails.

## Conclusions

Septic arthritis of the MSJ is a rare but important differential diagnosis in patients presenting with persistent anterior chest pain and chest wall swelling. Its uncommon presentation can lead to diagnostic delay, particularly when symptoms mimic cellulitis or more common cardiopulmonary conditions. In this case, the diagnosis was supported by clinical findings, elevated inflammatory markers, imaging features, multidisciplinary assessment, and response to antimicrobial therapy. Early cross-sectional imaging and multidisciplinary evaluation may facilitate timely diagnosis and management. Although surgical intervention may be required in complicated cases, carefully selected patients may respond successfully to conservative treatment alone.
